# A truncation mutant of adenomatous polyposis coli impairs apical cell extrusion through elevated epithelial tissue tension

**DOI:** 10.1002/cm.21893

**Published:** 2024-07-10

**Authors:** Wan J. Gan, Rabina Giri, Jakob Begun, Helen E. Abud, Edna C. Hardeman, Peter W. Gunning, Alpha S. Yap, Ivar Noordstra

**Affiliations:** ^1^ Centre for Cell Biology of Chronic Disease, Institute for Molecular Bioscience The University of Queensland St Lucia Queensland Australia; ^2^ Mater Research Institute The University of Queensland, Translational Research Institute Woolloongabba Queensland Australia; ^3^ Faculty of Medicine The University of Queensland St. Lucia Queensland Australia; ^4^ Department of Anatomy and Developmental Biology, Development and Stem Cells Program, Biomedicine Discovery Institute Monash University Clayton Victoria Australia; ^5^ Faculty of Medicine and Health, School of Biomedical Sciences University of New South Wales Sydney New South Wales Australia

**Keywords:** actin cytoskeleton, adherens junction, APC, apoptotic extrusion, tissue tension

## Abstract

Tissue tension encompasses the mechanical forces exerted on solid tissues within animal bodies, originating from various sources such as cellular contractility, interactions with neighboring cells and the extracellular matrix. Emerging evidence indicates that an imbalance in such forces can influence structural organization, homeostasis, and potentially contribute to disease. For instance, heightened tissue tension can impede apical cell extrusion, leading to the retention of apoptotic or transformed cells. In this study, we investigate the potential role of adenomatous polyposis coli (APC) in modulating tissue tension. Our findings reveal that expression of an APC truncation mutant elevates epithelial tension via the RhoA/ROCK pathway. This elevation induces morphological alterations and hampers apoptotic cell extrusion in cultured epithelial cells and organoids, both of which could be mitigated by pharmacologically restoring the tissue tension. This raises the possibility that APC mutations may exert pathogenetic effects by altering tissue mechanics.

## 1 INTRODUCTION

Solid tissues in animal bodies experience a variety of mechanical forces that can profoundly influence their form and function. This is well‐understood for mechanical tension (Chan et al., 2019; Eder et al., 2017; Harn et al., 2019), defined as forces that tend to pull tissues apart (Charras & Yap, 2018). Tissue mechanical tension guides cell behaviors such as proliferation, differentiation, and migration, thereby sculpting the mechanical characteristics and structural arrangement of tissues (Heer & Martin, 2017; Le & Mayor, 2023). Aberrant changes in tissue tension have been implicated in the pathogenesis of numerous diseases, including cancer, fibrosis, and cardiovascular disorders, highlighting its significance in disease progression and tissue pathology (Ayad et al., 2019; Zuela‐Sopilniak & Lammerding, 2022). It is therefore important to understand the mechanisms that determine tissue tension and how these influence morphogenesis and tissue homeostasis.

The physiologic and pathologic impact of tissue tension has been extensively investigated in epithelia. Here a key determinant of tissue tension arises from interactions between the actomyosin cytoskeleton and E‐cadherin adhesion at adherens junctions (AJ) (Campàs et al., 2023). The actomyosin network is the principal tension‐generator of cells; its actin filaments physically bind to the classical cadherin adhesion system; and E‐cadherin adhesions also regulate contractility by modulating cell signals, especially the RhoA GTPase (Ratheesh & Yap, 2012; Yap et al., 2018). Together, these cellular mechanisms allow actomyosin to generate mechanical tension at AJ in epithelial monolayers under steady‐state conditions. As well, junctional contractility influences homeostatic events, such as the process of apical extrusion where cells are expelled from a tissue apically, as part of tissue homeostasis or defense mechanism to remove damaged cells. Apical extrusion is elicited by a range of homeostatic changes in an epithelium including apoptosis and expression of oncogenes. In both cases, extrusion is driven by changes in actomyosin activity at the junctions between apoptotic or oncogene‐expressing cells and their immediate neighbors (Duszyc et al., 2021; Wu et al., 2014). Regulation of the AJ cytoskeleton then plays an important role in maintaining baseline tissue tension throughout a monolayer as well as the local changes necessary for apical extrusion.

Recently, we reported that the efficacy of apical extrusion is reduced when the pre‐existing mechanical tension of epithelial monolayers is elevated. This applied for both apoptotic (Mann et al., 2024) and oncogenic extrusion (Teo, Gomez, et al., 2020), suggesting that cytoskeletal regulators of contractility can influence the nexus between baseline tissue tension and apical extrusion. Adenomatous polyposis coli (APC), a well‐known oncogene which is best understood for its role as a regulator of the Wnt signaling pathway (Flanagan et al., 2021; Gao et al., 2002; Korinek et al., 1997; Morin et al., 1997; van Neerven et al., 2021), has been demonstrated to have the capacity to influence the cytoskeleton. It exerts such function through interactions with key regulators including mDia, EB1, and small GTPases, which play pivotal roles in modulating actomyosin contractility (Juanes et al., 2020; Kawasaki et al., 2000; Okada et al., 2010). Moreover, APC has been shown to participate in the regulation of AJ (Baro et al., 2023; Carothers et al., 2001). In this article, we investigate how cytoskeletal regulation by the APC gene product can influence apical extrusion through the modulation of tissue tension.

## 2 RESULTS AND DISCUSSION

### 2.1 An APC truncation mutant increases epithelial monolayer tension

**FIGURE 1:**
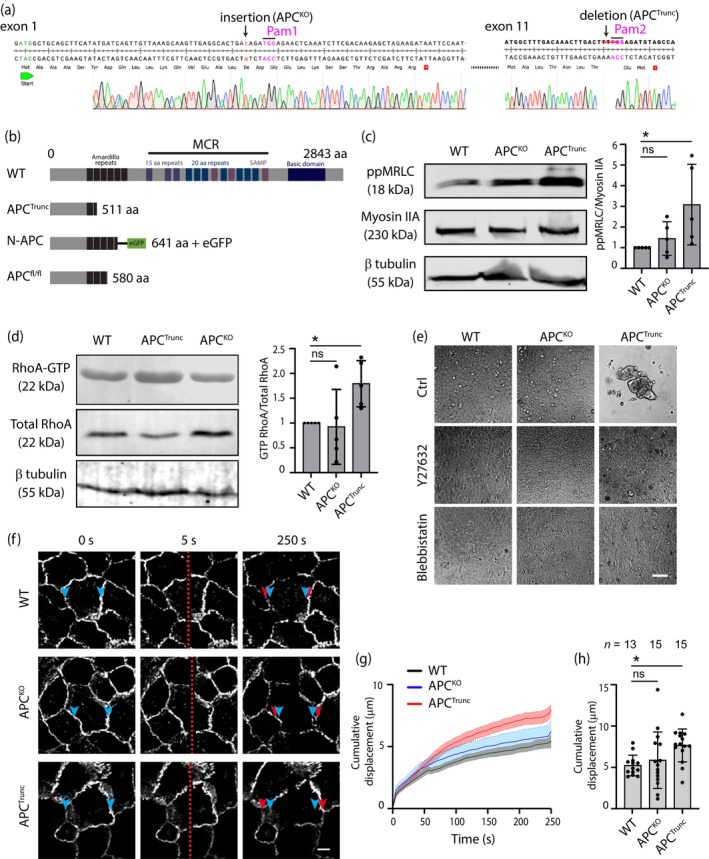
Truncation of adenomatous polyposis coli (APC) increases epithelial monolayer tension. (a) Genomic sequencing of human *APC* exons 1 and 11 from MCF10A APC^KO^ and APC^Trunc^ cells, respectively. (b) Illustration of APC full‐length protein (2843 amino acids; black: Amardillo repeats; purple: 15 aa repeats; green: 20aa repeats; pink: SAMP; navy: Basic domain), APC^Trunc^ (511 amino acids), N‐APC (641 amino acids) tagged with eGFP, and APC^fl/fl^ (580 amino acids). MRC, mutation region cluster. (c) Western blot analysis and quantification of wild type (WT), APC^KO^, and APC^Trunc^ cell extracts detected for phosphorylated myosin light chain (ppMRLC), myosin IIA, and β tubulin. *p = 0.0346; ns, not significant; Kruskal–Wallis test. Data are means ± SD, with individual data points indicated. (d) Western blot analysis and quantification of WT, APC^KO^, and APC^Trunc^ cell extracts detected for pull‐down RhoA‐GTP, total RhoA, and β tubulin. *N* = 5 independent experiments; ns, not significant; *p = 0.0167; Kruskal–Wallis test. Data are means ± SD, with individual data points indicated. (e) WT, APC^KO^, and APC^Trunc^ cells cultured on basement membrane extract type 2 (BME‐II)‐coated PDMS substrates (10–20 kPa). Cells were either untreated (Ctrl) or treated with 10 μM Y27632 or 10 μM Blebbistatin. Scale bar: 100 μm. (f) Live cell recording of tissue ablation with E‐cadherin 647 stained WT, APC^KO^, and APC^Trunc^ MCF10A cells. Blue arrow: cell border before ablation; red arrow: cell border after ablation; red scattered line: Laser ablation. Scale bar: 10 μm. (g) Cell border displacement of ablated WT (black), APC^KO^(blue), and APC^Trunc^ (red) over a time frame of 250 s. Data are means ± SEM, and are obtained from three independent experiments. Discrete quantification of total cell border displacement after ablation of WT, APC^KO^, and APC^Trunc^ monolayer from (f) and (g). *p = 0.0137; ns: not significant; Kruskal–Wallis test. Data are means ± SD, with individual data points indicated, and are obtained from 3 independent experiments.

Most colonic‐derived cell lines, such as Caco‐2, originate from colorectal cancer samples that express truncated APC. Therefore, to compare pathological truncations with full‐length APC, we used MCF10A cells, a breast‐derived epithelial cell line. This choice was guided by studies suggesting a potential association between APC mutations and breast cancer (Furuuchi et al., 2000; Moser et al., 1993; Woodage et al., 1998). To investigate the role of APC in regulating epithelial monolayer tension, we utilized CRISPR‐Cas9 technology to generate two distinct genetic modifications in MCF10A cells. First, we created a complete APC knockout (APC^KO^) by introducing a frameshift at amino acid 17, resulting in a premature stop at amino acid 30 (Figures 1a,b and S1a). Second, we induced a frameshift mutation in exon 11, leading to the expression of a truncated form of APC (APC^Trunc^; Figures 1a,b and S1a). This strategic alteration was guided by the recognition that a significant proportion of disease‐associated mutations in APC are found within the mutation cluster region, often leading to the expression of N‐terminal truncations of APC which contribute to pathological processes, such as gut polyp formation (Kawasaki et al., 2009; Kohler et al., 2008; Nakamura, 1995; Smith et al., 1993; Tominaga et al., 1998; Yonemura et al., 2010).

Nonmuscle Myosin II in the contractile apparatus is activated by phosphorylation of its regulatory light chain (MRLC) and generates force upon interaction with actin filaments (Krendel et al., 1999; Leerberg et al., 2014). To test if manipulation of APC affected actomyosin activity, we examined the phosphorylation status of MRLC by Western blot analysis. Cellular levels of ppMRLC, relative to Myosin IIA heavy‐chain expression, were not altered in the APC^KO^ cells compared with wild‐type (WT) controls. Strikingly, however, ppMRLC was elevated in the APC^Trunc^ cell line (Figure 1c), suggesting that expression of this N‐terminal mutant could stimulate actomyosin. To characterize this further, we then examined how APC manipulation affected morphology of these cell lines.

Previous studies have illustrated that cells seeded on soft substrates undergo notable morphological changes and roundup, particularly in response to heightened levels of tissue tension (Nyga et al., 2021; Pérez‐González et al., 2019). To investigate how increased tissue tension influences epithelial spreading on a soft substrate, we seeded cells onto basement membrane extract type 2 (BME‐II)‐coated polydimethylsiloxane (PDMS) layers with a stiffness ranging between 10 and 20 kPa (Teo, Lim, et al., 2020). While WT and APC^KO^ cells efficiently spread to form monolayers on the substrate (Figure 1e and Movie S1), APC^Trunc^ cells displayed a markedly different behavior. Rather than spreading, these cells appeared to roundup to form 3D aggregates (Figure 1e and Movie S1b). Interestingly, detailed time‐lapse imaging revealed that, after seeding, cells first spread but round up once the first cell–cell contacts are initiated (Figure S1, arrowhead). To test if this morphological pattern was due to increased contractility in the cells, we used either Y27632 to inhibit Rho kinase (ROCK), or blebbistatin to inhibit myosin II, both are principal contributors to actomyosin contractility. Both ROCK and myosin II inhibition reversed aggregation and rounding, allowing APC^Trunc^ cells to form a monolayer on the PDMS surface (Figure 1e and Movie S1c). However, this effect was reversible as cells lost their substrate connections and reverted to their rounded morphology upon Y27632 washout (Figure S1d and Movie S1d).

Together, these findings suggest that expression of APC^Trunc^ might increase actomyosin contractility via the RhoA‐ROCK pathway. To confirm this directly, we used pull‐down assays to measure cellular levels of active GTP‐RhoA. Indeed, GTP‐RhoA levels were significantly increased in the APC^Trunc^ cells compared with WT control cells (Figure 1d). We further examined tissue tension alterations in APC‐manipulated cell lines by assessing tissue recoil after laser ablation (Arnold et al., 2019). APC^Trunc^ monolayers demonstrated a significant increase in post‐ablation monolayer recoil compared with WT and APC^KO^ monolayers (Figure 1f–h and Movie S2). These findings indicate that the N‐terminal APC^Trunc^ mutant enhances RhoA signaling, leading to increased cell contractility and elevated tissue‐level mechanical tension.

### 2.2 The APC N‐terminus increases AJ tension

We then asked if the increased cell contractility was transmitted to AJ for mechanical tension. For this, we evaluated mechanosensitive conformational changes in α‐catenin within the E‐cadherin molecular complex. In the absence of mechanical tension, α‐catenin adopts a closed conformation, which masks epitopes in its central M‐domain and C‐terminal actin‐binding domain. Application of tension induces conformational changes that expose these epitopes, which can be detected by the α‐18 mAb and VD7 pAb, respectively (Duong et al., 2021; Noordstra et al., 2023; Yonemura et al., 2010). Accordingly, we used these markers of molecular‐level tension to test if AJ tension was altered by manipulation of APC. Cells were grown to confluence on glass coverslips to facilitate the formation of epithelial monolayers, then fixed and immunostained for α‐catenin and its epitopes.

**FIGURE 2:**
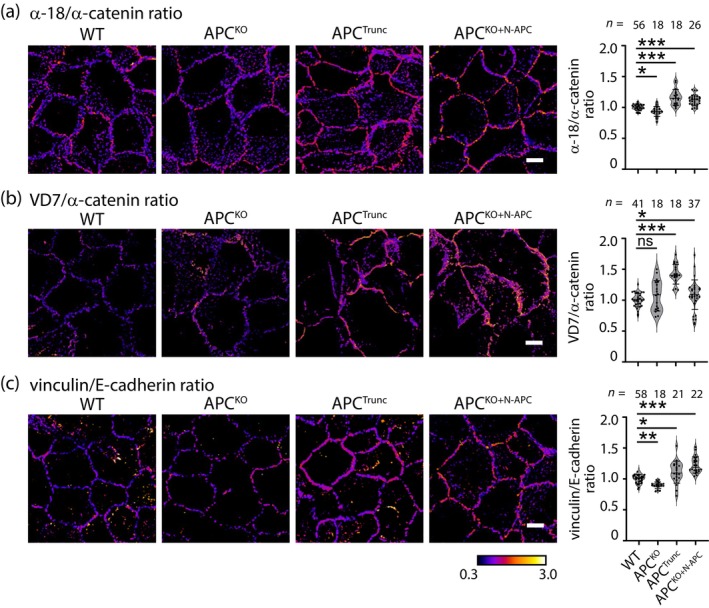
The adenomatous polyposis coli (APC) N‐terminus increases adherens junction tension. (a–c) Ratiometric images and quantifications of wild type (WT), APC^KO^, APC^Trunc^, and APC^KO+N‐APC^ MCF10A cells stained for (a) α‐18/α‐catenin, (b) VD7/α‐catenin, or (c) vinculin/E‐cadherin respectively. Scale bar: 10 μm. *p < 0.05; **p < 0.01; ***p < 0.001; ns, not significant; Kruskal–Wallis test. Data are means ± SD, with individual data points indicated, and are obtained from three independent experiments.

Interestingly, we observed intensified labelling with both α‐18 and VD7 antibodies in APC^Trunc^ cells compared with WT and APC^KO^ cells (Figure 2a,b). This supports the notion that the increased tension is indeed reflected by a conformational change of α‐catenin. To confirm that this mechanical change was due to the N‐terminal fragment of APC, we exogenously reintroduced a green fluorescent protein (GFP)‐tagged truncation of APC, similar to APC^Trunc^, in the APC^KO^ cells (Figures 1b and S1b). These cells were designated as APC^KO+N‐APC^. Consistent with the increased junctional tension observed in APC^Trunc^, the APC^KO+N‐APC^ monolayer exhibited an increase in α‐18 and VD7 labelling as compared with the WT and APC^KO^ cells (Figure 2a,b).

As a further test of tension at AJ, we immunostained for vinculin. Vinculin binds to the central domain of α‐catenin when its conformation is opened on application of tension (Yonemura et al., 2010). Consistent with our previous findings, junctional vinculin labelling intensified in APC^Trunc^ and APC^KO+N‐APC^ cells compared with WT and APC^KO^ cells (Figure 2c). Altogether, our data indicate that baseline mechanical tension at AJ is increased when expression of N‐terminal APC mutants stimulates contractility.

### 2.3 The APC N‐terminus compromises apical extrusion of apoptotic cells

We recently reported that tissue mechanical hypertension disrupted the ability of epithelial monolayers to eliminate apoptotic cells by apical extrusion (Mann et al., 2024). Therefore, we tested whether the tissue mechanical changes induced by APC^Trunc^ mutants also affected apoptotic extrusion.

**FIGURE 3:**
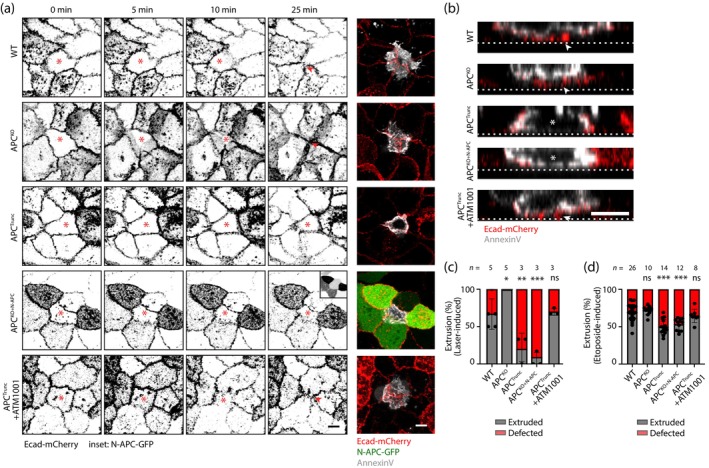
Expression of the adenomatous polyposis coli (APC) N‐terminus compromises apical extrusion of apoptotic cells. (a) Live cell recording of apoptotic cell extrusion within E‐cadherin‐mCherry (red) expressing wild type (WT), APC^KO^, APC^Trunc^, APC^KO+N‐APC^ and ATM1001‐treated APC^Trunc^ MCF10A cells. N‐APC‐GFP expression (green). Annexin V cell death marker (white). *cell injured with two‐photon pulsed laser; red arrow: closure of the epithelial layer upon extrusion. Scale bar: 10 μm. (b) Orthogonal views of cells in (a) at 25 min *retained cells after laser injury; red arrow: closure of the epithelial layer upon extrusion; white‐dotted line: glass surface. Scale bar: 10 μm. (c) Quantification of apical cell extrusion as a result of laser injury as in (a) and (b). *p < 0.05; **p < 0.01; ***p < 0.001; ns, not significant; Kruskal–Wallis test. Data are means ± SD, with individual data points indicated, and are obtained from three to five independent experiments. (d) Quantification of apical cell extrusion as a result of etoposide treatment. ***p < 0.001; ns, not significant; Kruskal–Wallis test. Data are means ± SD, with individual data points indicated, and are obtained from three independent experiments.

We utilized E‐cadherin tagged with mCherry to delineate individual cells within the monolayer. To induce apoptosis and monitor extrusion, we selectively injured the nuclei of single cells using a two‐photon pulsed laser beam and subsequently imaged the cells for 1 h following cell death. Any extrusion that was not completed within the 1‐h window was classified as defective. In WT monolayers, ~67% of the injured cells were extruded upon laser injury (Figure 3a–c and Movie S3). The extrusion process was characterized by the apical expulsion of the apoptotic cell (identified with Annexin V) and the simultaneous extension of its neighbors to form a rosette that seals the epithelium (Figure 3a,b). APC^KO^ monolayers were able to efficiently extrude apoptotic cells with an apical extrusion rate that was greater than WT cells (Figure 3a–c and Movie S3b). This increase in extrusion efficacy may be due to altered adhesive properties (Carothers et al., 2001; Mastrogiovanni et al., 2022), potentially making it easier for the cells to disengage from basal connections and be expelled from the epithelial layer.

In contrast, extrusion was markedly compromised in APC^Trunc^ cells, with extrusion rates falling below 20% (Figure 3a–c and Movie S3c). This was attributable to the APC truncation fragment, as the capacity of APC^KO^ monolayers to support apical extrusion was significantly compromised by expression of the APC N‐terminal transgene (APC^KO+N‐APC^, Figure 3a–c and Movie S3d). To test if aberrant tissue tension was responsible for this defect in apoptotic extrusion, we used the ATM1001 tropomyosin inhibitor, which reduces junctional contractility by disrupting the binding of tropomyosin 3.1 to actin filaments (Kee et al., 2018; Mann et al., 2024). Remarkably, a 24‐h treatment with 2.5 μM ATM1001 effectively restored the apoptotic extrusion rate in the APC^Trunc^ monolayer to levels comparable to those observed in the WT cells (Figure 3a–c and Movie S3e).

As an alternative test, we investigated extrusion dynamics upon apoptosis induced with the podophyllotoxin etoposide (Karpinich et al., 2002). No difference was observed between WT and APC^KO^ cells. Nonetheless, APC^Trunc^ and APC^KO+N‐APC^ exhibited reduced extrusion as compared with the WT and APC^KO^, and the reduction was again reverted by the addition of ATM1001. The data closely paralleled our observations from the laser‐induced apoptosis, underscoring the suppression of cellular extrusion under conditions of elevated contractility and tissue tension (Figure 3d).

### 2.4 APC N‐terminus‐induced increase in tension compromises apoptotic extrusion in intestinal organoid monolayers

**FIGURE 4:**
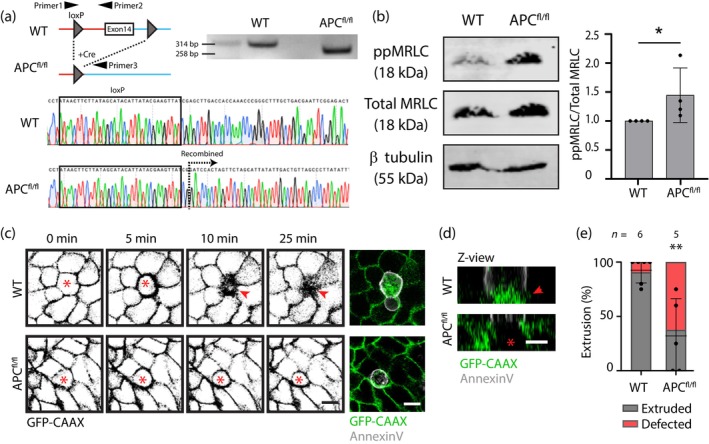
Adenomatous polyposis coli (APC) N‐terminus‐induced increase in tension compromises apoptotic extrusion in intestinal organoid monolayers. (a) Illustration of in vitro Cre recombination resulting in the deletion of *APC* exon 14, causing a genomic DNA PCR product shift from 314 to 258 bp. Sanger sequencing further confirmed recombination. Western blot analysis of WT and APC^fl/fl^ organoid extracts detected for phosphorylated myosin light chain (ppMRLC), total MRLC, and β tubulin. *p = 0.0286; Mann–Whitney test. Data are means ± SD, with individual data points indicated. (c) Live cell recording of apoptotic cell extrusion within GFP‐CAAX (green) expressing wild type (WT) and APC^fl/fl^ organoid monolayers. Annexin V cell death marker (white). *Cell injured with two‐photon pulsed laser; red arrow: closure of the epithelial layer upon extrusion. Scale bar: 10 μm. (d) Orthogonal views of organoid monolayers in (c) at 25 min. *Retained cells after laser injury; red arrow: closure of the epithelial layer upon extrusion. Scale bar: 10 μm. (e) Quantification of apical cell extrusion as a result of laser injury as in (c,d). **p < 0.01; Mann–Whitney *U* test. Data are means ± SD, with individual data points indicated, and are obtained from five to six independent experiments.

Finally, we used mouse intestinal organoids to corroborate the results of our genome‐edited cell lines. Small intestinal organoids were derived from APC^580^ mice, featuring loxP sites precisely integrated around APC exon 14 (Shibata et al., 1997). Exon 14 was then excised by in vitro Cre‐recombination, resulting in the generation of truncated APC akin to the APC^Trunc^ MCF10A model utilized in our study (Figures 1b and 4a). Henceforth, we will refer to the APC‐mutated organoids as APC^fl/fl^.

Western blot analysis revealed that APC^fl/fl^ organoids demonstrated elevated ppMRLC compared with the WT counterparts (Figure 4b), indicating increased tissue contractility within the APC^fl/fl^ organoids, mirroring what we observed with the APC^Trunc^ MCF10A model. Then, we investigated whether cell extrusion is impaired within the APC^fl/fl^ organoids. To test this, we cultured organoids as 2D monolayers, using the protocol of Xi et al. (2022). Dissociated organoids were seeded onto cross‐linked BME gels with a stiffness of ~400 Pa, thereby facilitating optimal growth of intestinal organoids as 2D monolayers (Xi et al., 2022). Employing this method, we observed a reduction in cell extrusion upon laser injury to 37% within the APC^fl/fl^ monolayer, compared with 90% in the WT monolayer (Figure 4c–e and Movie S4). These findings support the data from MCF10A cells, confirming that cellular extrusion is impeded under conditions of APC N‐terminus‐induced elevation in tissue contractility. This further suggests that APC^Trunc^ has a capacity to inhibit apical extrusion by elevating tissue tension, which can operate in different epithelial tissues, highlighting the generality of our findings.

Overall, our study suggests a model wherein an N‐terminal APC truncation induces heightened tissue tension, resulting in morphological alterations and compromised apical cell extrusion within epithelial monolayers. From a clinical perspective, both phenotypes may synergistically foster the onset of pathological conditions. Morphological transformations have been observed in other oncogenic transformed cells (Nyga et al., 2021; Pérez‐González et al., 2019), suggesting a potentially universal feature of tumor formation. Furthermore, the impairment of apical extrusion following APC truncation‐induced alteration of tissue tension may allow the accumulation of aberrant cells within the tissue (Teo, Gomez, et al., 2020; Wee et al., 2020). These cells can then proliferate, forming a population capable of undergoing structural transformation into polyps or tumors.

Central to this mechanism is the critical role of RhoA in regulating epithelial tension by modulating the actomyosin cytoskeleton. Upon activation, RhoA triggers the downstream activation of ROCK, which enhances actomyosin contractility by phosphorylating MRLC and inhibiting myosin light chain phosphatase (MLCP; Kimura et al., 1996; Leerberg et al., 2014). Our findings demonstrate that the expression of the N‐terminal APC truncation mutant indeed amplifies tissue tension via the RhoA‐ROCK pathway. Significantly, inhibition of cell contractility by the orthogonal strategy of inhibiting tropomyosin effectively restores tissue tension, reverses morphological changes, and rescues apoptotic cell extrusion.

A key question for future research is to elucidate how expression of APC^Trunc^ stimulates RhoA signaling within cells. One possibility is that expression of this APC mutant activates a RhoA Guanine nucleotide exchange factor (GEF). The Armadillo repeats region within the N‐terminus of APC has been reported to activate the GEF Asef (Kawasaki et al., 2000; Kawasaki et al., 2009). Asef, in turn, has been demonstrated to activate Rac1, CDC42, and also RhoA (Gotthardt & Ahmadian, 2007; Kawasaki et al., 2000; Kim et al., 2018). Full‐length APC undergoes a self‐folding mechanism that auto‐inhibits its N‐terminus, thereby regulating Asef activation (Yang et al., 2021). However, the truncated N‐terminus has been demonstrated to effectively activate Asef (Kawasaki et al., 2000). Another possibility involves the activation of Wnt pathways, which may in turn activate RhoA through the Dsh and Daam1 mediated signaling cascade. Understanding whether Asef, Daam1 or another RhoA GEF is activated by APC^Trunc^ is crucial for elucidating how this APC mutation impacts epithelial tissue mechanics and homeostasis. Our findings underscore the importance of considering APC‐mediated regulation of tissue tension as a contributing factor to disease pathology.

## 3 MATERIALS AND METHODS

### 3.1 Cell culture

MCF10A was maintained in DMEM/F12 (Invitrogen; catalog no. 11330‐032) containing 5% heat‐inactivated horse serum (Invitrogen; catalog no. 16050‐122), 20 ng/mL human epidermal growth factor, 0.5 mg/mL hydrocortisone, 100 ng/mL cholera toxin, 10 μg/mL insulin, and 100 U/mL penicillin/streptomycin under standard cell culture condition (37°C, 95/5% air/CO_2_).

Small intestinal organoids were harvested from the APC^580s^ mouse as described before (O'Rourke et al., 2016; Shibata et al., 1997). The organoids were encapsulated in 50% Cultrex BME‐II (R&D Systems; catalog no. 3533‐010‐02) and maintained in advanced DMEM/F12 (Invitrogen; catalog no. 12634‐010) supplemented with 50% medium conditioned with Wnt‐3A, R‐spondin 3 and noggin, 20% fetal bovine serum (FBS), and 100 U/mL penicillin/streptomycin. In total, 10 μM of Y27632 (Watanabe et al., 2007) and 10 μM of SB431542 (Miyoshi et al., 2012) were supplemented for the first 24 h after passaging to increase viability, followed by changing to 2 μM of IWP‐2 and 2 mM of valproic acid (Yin et al., 2014) and culture for 3 days for the enterocyte differentiation.

### 3.2 APC deletion

APC knockout (APC^KO^) and truncated (APC^Trunc^) MCF10A cells were generated through CRISPR/Cas9‐mediated gene modification. sgRNAs targeting APC exon 1 5′‐GCAAGTTGAGGCACTGAAGA‐3′ and exon 11 5′‐GCTTTGACAAACTTGACTTT‐3′ (Saito‐Diaz et al., 2018) were obtained from Integrated DNA Technologies. The sequences were cloned into pSpCas9 (BB)‐2A‐Puro (PX459) vector (Addgene plasmid no. 62988), followed by lipofectamine‐mediated transfection into the MCF10A cells. Cells were then selected in 1 μg/mL puromycin for 48 h and single clones were sorted through flow cytometry. Genotypes of the single clones were confirmed by polymerase chain reaction (PCR) and Sanger sequencing of *APC* exons 1 and 11 using the following primers: exon 1: 5′‐CTTATAGGTCCAAGGGTAGCCAAG‐3′ and 5′‐TAAAAATGGATAAACTACAATTAAAAG‐3′; exon 11: 5′‐GATGATTGTCTTTTTCCTCTTGC‐3′ and 5′‐CTGAGCTATCTTAAGAAATACATG‐3′.

APC^fl/fl^ small intestinal organoids were generated through Cre‐loxP‐mediated recombination by adding TAT‐Cre recombinase in vitro to dispersed organoids for 8 h, followed by encapsulating the organoids back into BME‐II gel (Chan et al., 2023). The organoids were then cultured in nonconditioned medium for at least a week to select the clusters with successful recombination. Successful recombination was confirmed by gel electrophoresis and Sanger sequencing of the genomic DNA PCR product using the following primers: 5′‐GTTCTGTATCATGGAAAGATAGGTGGTC‐3′, 5′‐CACTCAAAACGCTTTTGAGGGTTGATTC‐3′, and 5′‐GAGTACGGGGTCTCTGTCTCAGTGAA‐3′ (Shibata et al., 1997).

### 3.3 Viral transduction

Stable cell lines expressing E‐cadherin‐mCherry, APC^KO^ cells expressing N‐APC‐GFP (APC^KO+N‐APC^), and organoids expressing eGFP‐CAAX were generated through lentiviral transduction. Lentiviral construct pLL5.0 E‐cadherin shRNA/mE‐cadherin‐mCherry (Addgene plasmid no. 101282), pLL5.0‐eGFP‐CAAX (Addgene plasmid no. 187252) or pLL5.0‐N‐APC‐GFP, together with third‐generation lentiviral packaging vectors (Addgene plasmids nos. 12251, 12253, and 12259) were transfected into HEK293T cells to generate viral particles. Secreted lentivirus was collected at Days 2 and 3 posttransfection. After addition of polyethylene glycol 6000 (PEG 6000), virus was precipitated by centrifugation at 1500*g* for 1 h at 4°C. Next, the virus was added to target cells or dissociated organoids and incubated 48 or 24 h, respectively, in the presence of 10 μg/mL polybrene. mCherry‐positive cells were selected through flow cytometry, whereas GFP‐positive organoids were manually selected using a stereomicroscope.

### 3.4 PDMS generation

Equal amount of Cy‐A and Cy‐B (Dow Corning) were mixed in a 50 mL tube. Approximately 0.56 grams of the PDMS mixture was added onto a 35 mm glass bottom dish, spin coated at 500 rpm for 30 s, and cured at 80°C for 2 h. Cured PDMS was coated with 5% BME‐II gel overnight at 4°C, followed by incubation in 1% pluronic acid for 30 min. Dish was thoroughly washed with phosphate‐buffered saline (PBS) before cell seeding.

### 3.5 Western blotting

Cells grown to full confluency within a 6‐well plate or organoids isolated from the BME‐II gel were lysed using lysis buffer (50 mM Tris‐Cl, 2% sodium dodecyl sulfate, 0.1% bromophenol blue, 10% glycerol, 0.04 M DTT, and 1×phosSTOP). After heating the lysate at 98°C for 10 min, the proteins were separated on polyacrylamide gels. The proteins were then transferred onto methanol‐activated polyvinylidene difluoride membranes, followed by blocking with 5% skim milk for 1 h at room temperature. Next, the membranes were incubated in primary antibodies (rabbit polyclonal antibody against human APC (Cell Signaling; catalog no. 2504), rabbit polyclonal antibody against ser19‐phospho‐specific myosin light chain (Chemicon; catalog no. AB3381), mouse monoclonal antibody against ser19‐phospho‐specific myosin light chain (Cell Signaling; catalog no. 3675), rabbit monoclonal antibody against myosin light chain 2 (Cell Signaling; catalog no. 8505), or rabbit polyclonal antibody against nonmuscle myosin II heavy chain A [BioLegend; catalog no. PRB‐440P]) diluted in 5% bovine serum albumin (BSA) in tris‐buffered saline (TBS) overnight at 4°C. The blots were then washed and probed with secondary antibodies (Goat anti‐mouse IRDye 680RD or goat anti‐rabbit IRDye 800CW) diluted in 5% skim milk for 1 h at room temperature. After washing, the blots were visualized using a LI‐COR Odyssey CLx system.

### 3.6 Immunofluorescence staining

Cells grown to full confluency on coverslips were prepermeabilized in cytoskeleton stabilization buffer (10 mM PIPES, 50 mM NaCl, 3 mM MgCl_2_, 300 mM sucrose, 1× protease inhibitor) containing 0.5% triton X‐100 for 5 min on ice, followed by fixation in 4% paraformaldehyde diluted in PBS at room temperature for 10 min. After washing three times with PBS, the fixed cells were blocked in 3% BSA for 1 h at room temperature and incubated in primary antibodies (rat monoclonal antibody against α‐catenin M‐domain [α18, gift from Dr A. Nagafuchi, Kumamoto University, Japan]; mouse monoclonal antibody against α‐catenin [Invitrogen; catalog no. 13‐9700]; Rabbit polyclonal antibody against α‐catenin ABD [VD7, gift from Dr D. Vestweber, Max Planck Institute for Molecular Biomedicine, Germany]; rabbit polyclonal antibody against vinculin [Abcam; catalog no. ab91459]; rat monoclonal antibody against E‐cadherin [Zymed; catalog no. 13‐1900]) overnight at 4°C. Cells were washed three times with PBS, followed by incubation in secondary antibodies (goat antibodies conjugated with Alexa Fluor 488, 546, or 647 [Invitrogen]) for 1 h at room temperature. Finally, cells were washed three times with PBS and mounted onto glass slides with Prolong gold antifade mountant (Invitrogen; catalog no. P36930). Imaging was performed using a Zeiss LSM880 confocal microscope.

### 3.7 Monolayer laser ablation

Cells were grown on 35 mm glass bottom dishes to 90% confluency. Cells were preincubated with mouse antihuman E‐cadherin ectodomain antibody conjugated with Alexa Fluor 647 (BD Bioscience; catalog no. 563571), diluted 1 in 200 dilution in Hank's balanced salt solution (Sigma‐Aldrich; catalog no. H8264) supplemented with 5% FBS, 15 mM 4‐(2‐hydroxyethyl)‐1‐piperazineethanesulfonic acid (HEPES), 5 mM CaCl_2_, and 10 mM glucose for 30 min prior to imaging. Using a Zeiss LSM710 confocal microscope equipped with a MaiTai eHP tuneable 760–1040 nm laser, the monolayer was ablated over a region of 0.53 μm (width) × 79.07 μm (height) with a 790 nm laser (70% laser power, 50 iterations), followed by recording of the monolayer recoil for 250 s at an interval of 5 s. Cumulative displacement of recoil was calculated by measuring the distance at the final time point after ablation (in between the red arrows, Figure 1f), minus the distance at the time point before ablation (in between the blue arrows, Figure 1f).

### 3.8 Laser injury

Cells were grown on 35 mm glass bottom dishes to 90% confluency and imaged in Hank's balanced salt solution (Sigma‐Aldrich; catalog no. H8264) supplemented with 5% FBS, 15 mM HEPES, 5 mM CaCl_2_, and 10 mM glucose. For the tropomyosin inhibition, cells were incubated with 2.5 μM ATM1001 for 24 h and treatment was maintained throughout imaging.

Organoids were harvested from BME‐II gels by adding cold trypLE solution (Gibco; catalog no. 12604013) to the gels. The organoids were further dissociated in the solution by incubating for 3 min at 37°C, followed by pipetting up and down 30 times. The dissociated organoids were then seeded onto BME‐II gel cross‐linked with N‐hydroxysuccinimide and 1‐ethyl‐3‐(3‐dimethylaminopropyl)carbodiimide (Xi et al., 2022), and cultured as 2D monolayers until confluent.

Imaging cells and organoid monolayers were performed on a Zeiss LSM710 confocal microscope equipped with a MaiTai eHP tuneable 760–1040 nm laser. The nuclei of the cells were ablated with a 790 nm laser (70% laser power, 30 iterations) and the process of extrusion was recorded for 1 h at an interval of 30 s. Apoptosis of the injured cells was confirmed using Annexin V conjugated with Alexa Fluor 647 (Thermo Fisher Scientific; catalog no. A23204).

### 3.9 Etoposide assay

Cells grown to full confluency were incubated with 500 μM etoposide (Adooq Biosciences LLC no. A10373) for 6 h at 37°C. Next, cells were fixed with 4% paraformaldehyde diluted in PBS at room temperature for 10 min and permeabilized with 0.1% triton X‐100 for 10 min, followed by immunofluorescence staining. Caspase 3 (Cell signaling; catalog no. 9661) was used to identify the apoptotic cells and E‐cadherin mAb antibody (Zymed; catalog no. 13‐1900) was used to highlight the borders of individual cells. Imaging was performed on a Zeiss Axio Observer 7 microscope using a C‐Apochromate 40/1.20 water objective over a Z stack of 11 μm. Caspase 3‐positive cells displaying an enclosed E‐cadherin signal underneath were classified as extruded, otherwise cells were classified as nonextruded. Examples of cells classified as extruded (from WT monolayer) or nonextruded (from APC^Trunc^ monolayer) are provided in Figure S1e. The data were quantified and presented as a percentile in which the number of extruded/nonextruded cells was divided by the total number of cells positive for Caspase 3.

### 3.10 GTP‐RhoA pulldown

Pulldown of GTP‐RhoA was performed according to the manufacturer's instruction (Cytoskeleton; catalog no. BK030). In brief, cells were serum‐starved for 24 h, followed by serum stimulation for 30 min. Next, cells were washed with ice‐cold PBS, lysed, and incubated with 15 μL Rhotekin‐RBD affinity beads for 2 h at 4°C with rotation. The beads were then collected and washed. Bound GTP‐RhoA was extracted by heating the beads in western blot lysis buffer for 5 min and the lysates were analyzed by western blot.

### 3.11 Image and statistical analysis

Images were analyzed using FIJI (ImageJ). Immunofluorescent images were Z‐projected as the sum of intensity. Junctional marker (α‐catenin or E‐cadherin) signals were used to create a mask after a Gaussian blur was applied. The mask was projected onto each channel to obtain average intensities of the various stainings.

Ratiometric measurements were obtained by dividing the average intensities of the tension‐sensitive protein by the average intensities of the total junctional protein. Ratios were further normalized against the WT control.

For laser injury, cells were classified as extruded if the neighboring cells successfully closed the gap underneath the Annexin V‐positive injured cell within 1 h of imaging. Otherwise, the extrusion was classified as noncompleted. The data were quantified and presented as a percentile in which the number of extruded/nonextruded cells was divided by total number of cells recorded.

Statistical analysis was performed using GraphPad Prism. Statistical parameters for individual experiments are listed in the corresponding figure legends. This includes the statistical test performed, sample size (*n*), number of independent experiments, and statistical significance.

## AUTHOR CONTRIBUTIONS

The study is conceptualized by W.J.G., A.S.Y., and I.N. W.J.G. performed all the experiments and analysis under the supervision of A.S.Y. and I.N. R.G., J.B., and H.E.A. provided advice for the works related to organoids. E.C.H. and P.G. provided the tropomyosin inhibitor ATM1001 used in the study.

## CONFLICT OF INTEREST STATEMENT

E.C. Hardeman reports receiving a commercial research grant from and has ownership interest (including patents) in TroBio Therapeutics Pty Ltd. P.W. Gunning reports receiving other commercial research support from and has ownership interest (including patents) in TroBio Therapeutics Pty Ltd.

## Supporting information


**Figure S1.** (a) Western blot of WT, APC^KO^, and APC^Trunc^ cell extracts detected for full‐length APC and β tubulin. (b) Western blot of WT and APC^KO+N‐APC^ cell extracts detected for GFP and β tubulin. (c) Live cell recording of APC^Trunc^ cells on BME‐II‐coated PDMS substrates (10–20 kPa). Arrowhead indicates the initiation of cell–cell contact. Scale bar: 50 μm. (d) Live cell recording of APC^Trunc^ monolayer after washing out the Y27632. Scale bar: 100 μm. Scale bar of the zoom out image: 500 μm. (e) Example of extruded cell and nonextruded cell from the WT and APC^Trunc^ monolayer respectively after etoposide‐induced cell death. Red: E‐cadherin; Green: Caspase 3; blue: DAPI. Scale bar: 10 μm.

**Movie S1.** Phase contrast live cell imaging of (a) WT, (b) APC^Trunc^, (c) Y27632‐treated APC^Trunc^, and (d) Y27632 wash off APC^Trunc^ MCF10A cells related to Figure 1. Scale bar: 50 μm.

**Movie S2.** Laser ablation of E‐cadherin 647 antibody stained (a) WT, (b) APC^KO^, (c) APC^Trunc^ monolayer. Scale bar: 10 μm.

**Movie S3.** Laser‐induced apoptotic cell extrusion of E‐cadherin‐mCherry (red) expressing (a) WT, (b) APC^KO^, (c) APC^Trunc^, (d) APC^KO+N‐APC^, and (e) ATM1001‐treated APC^Trunc^ MCF10A cells related to Figure 3. Green: N‐APC‐GFP expression; White: Annexin V cell death marker. Scale bar: 10 μm.

**Movie S4.** Laser‐induced apoptotic cell extrusion of GFP‐CAAX (green) expressing (a) WT, and (b) APC^fl/fl^ organoid monolayers related to Figure 4. White: Annexin V cell death marker. Scale bar: 10 μm.

## Data Availability

The data that support the findings of this study are available from the corresponding author upon reasonable request.
