# Supplementary material for: A truncation mutant of adenomatous polyposis coli impairs apical cell extrusion through elevated epithelial tissue tension
Source: Cytoskeleton (Hoboken). 2024 Jul 10;82(7):444–55. doi: 10.1002/cm.21893 (PMC12247696; doi:10.1002/cm.21893)
Supplement: Supplementary file 1 — Figure S1. (a) Western blot of WT, APCKO, and APCTrunc cell extracts detected for full‐length APC and β tubulin. (b) Western blot of WT and APCKO+N‐APC cell extracts detected for GFP and β tubulin. (c) Live cell recording of APCTrunc cells on BME‐II‐coated PDMS substrates (10–20 kPa). Arrowhead indicates the initiation of cell–cell contact. Scale bar: 50 μm. (d) Live cell recording of APCTrunc monolayer after washing out the Y27632. Scale bar: 100 μm. Scale bar of the zoom out image: 500 μm. (e) Example of extruded cell and nonextruded cell from the WT and APCTrunc monolayer respectively after etoposide‐induced cell death. Red: E‐cadherin; Green: Caspase 3; blue: DAPI. Scale bar: 10 μm. [file CM-82-444-s002.pdf]

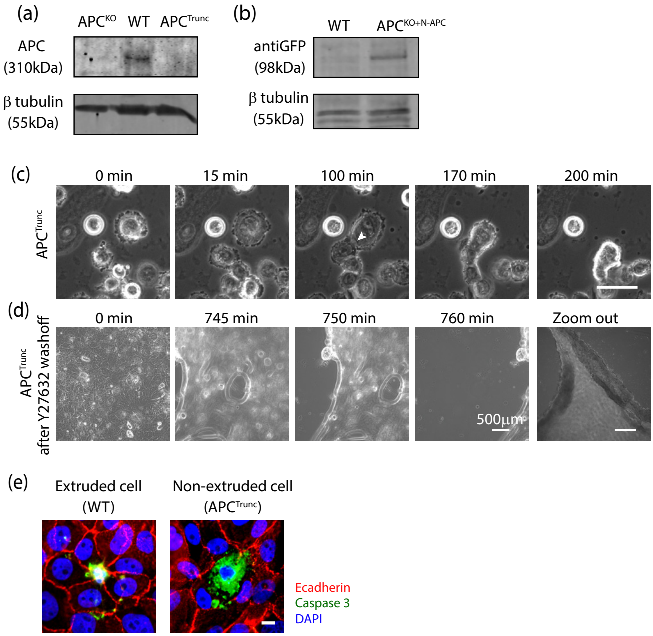

Supplementary figure 1:

(a) Western blot of WT, APC^{KO}, and APC^{Trunc} cell extracts detected for full-length APC and β tubulin.

(b) Western blot of WT and $APC^{KO+N\text{-}APC}$ cell extracts detected for GFP and β tubulin.

(c) Live cell recording of APC^{Trunc} cells on BME-II-coated PDMS substrates (10-20kPa). Arrowhead indicates the initiation of cell-cell contact. Scale bar: 50μm.

(d) Live cell recording of APC^{Trunc} monolayer after washing out the Y27632. Scale bar: 100μm. Scale bar of the zoom out image: 500μm.

(e) Example of extruded cell and non-extruded cell from the WT and APC^{Trunc} monolayer respectively after etoposide-induced cell death. Red: E-cadherin, Green: Caspase 3, Blue: DAPI. Scale bar: 10μm.
